# Factors associated with awareness, treatment and control of hypertension among 3579 hypertensive adults in China: data from the China Health and Nutrition Survey

**DOI:** 10.1186/s12889-021-10417-4

**Published:** 2021-03-01

**Authors:** Junxiang Wei, Yang Mi, Yan Li, Bo Xin, Youfa Wang

**Affiliations:** 1 Department of Obstetrics, Northwest Women’s and Children’s Hospital, Xi’an, 710061 Shaanxi China; 2 Global Health Institute, Xi’an Jiaotong University Health Science Center, Xi’an, Shaanxi China; 3 Department of Epidemiology and Biostatistics, School of Public Health, Xi’an Jiaotong University Health Science Center, Xi’an, Shaanxi China; 4 Department of Population Health Science and Policy, Icahn School of Medicine at Mount Sinai, New York, NY USA

**Keywords:** Hypertension, Awareness, Treatment, Control, China

## Abstract

**Background:**

The prevalence of hypertension is high and rising in China, but most people with hypertension do not have their blood pressure under control. This study investigated hypertension awareness, treatment, and control and their associated factors among Chinese adults.

**Methods:**

Data collected from the 2011 China Health and Nutrition Survey (CHNS) from 12,991 Chinese adults were used. Hypertension was defined as systolic blood pressure ≥ 140 mmHg, diastolic blood pressure ≥ 90 mmHg, self-reported prior diagnosed hypertension, or taking antihypertensive medications. Hypertension awareness, treatment, and control were defined as a self-reported diagnosis of hypertension, current use of antihypertensive medication, and blood pressure < 140/90 mmHg, respectively. Multivariate logistic regression was performed to examine factors associated with hypertension awareness, treatment, and control.

**Results:**

Overall, 3579 (27.6%) of the CHNS adult participants had hypertension, of whom 55.7% were aware of their diagnosis, 46.5% were treated with antihypertensive medications, but only 20.3% had their blood pressure under control. Higher hypertension treatment was associated with older age (OR = 2.57; 95%CI, 1.65–4.02), urban residency (1.50; 1.14–1.97), living in the Eastern region (1.52; 1.14–2.01), and being overweight/obese (1.99; 1.39–2.84). Hypertension awareness was associated with similar factors as hypertension treatment but was also associated with being female (1.37, 1.12–1.66). Poor hypertension control was associated with being overweight/obese (0.56; 0.42–0.76) and minority ethnicity (0.52; 0.31–0.86).

**Conclusion:**

Hypertension is a major public health challenge in China. The prevalence of hypertension awareness, treatment, and control are still low despite existing public health policies and programs to reduce the burden of hypertension. More intensive hypertension screening and treatment programs are warranted in China.

## Background

Hypertension is one of the most prevalent chronic diseases in many countries and is the most common modifiable risk factor for other severe chronic conditions such as heart disease, stroke, kidney disease, and subsequent morbidity and mortality [1]. It leads to 9.4 million deaths each year worldwide, with the condition disproportionately affecting low- and middle-income countries [2, 3].

China has the largest population with hypertension in the world with an estimated 244.5 million (23.2%) adults having hypertension in 2015 [4]. Recent population-based surveys estimated that almost one third of Chinese adults have hypertension [5–7]. As the society ages and the prevalence of unhealthy lifestyles (e.g., high sodium consumption, physical inactivity, binge drinking) continue to increase, hypertension has become a major threat to the improvement of population health ( [8, 9]. Effective hypertension prevention programs and timely treatment and control are critical to alleviate the burden of hypertension.

Progress has been made to increase the awareness, treatment, and control rates of hypertension in China over the past two decades [9, 10]. For example, there is an increase in health insurance coverage and utilization of healthcare resources as well as a wide implementation of evidence-based blood pressure management programs [11, 12], all of which have improved hypertension prevention and management in the country. However, China still underperforms in hypertension control compared to many other countries [7].

In this study, we aimed to: (a) examine the prevalence of hypertension awareness, treatment, and control among Chinese adults overall and by age, sex, region, and socioeconomic status, (b) compare the prevalence results of this study with those from the previous two similar studies, and (c) describe factors that are associated with hypertension awareness, treatment, and control.

## Methods

### Data source

We used data from the 2011 China Health and Nutrition Survey (CHNS), which is an ongoing, large-scale, population-based survey initiated in 1989. CHNS used a stratified multistage, random cluster sampling strategy to select participants from 288 communities across a large number of primary provinces/autonomous cities [13]. We have considered the effect of this complex sampling strategy in the analysis. The CHNS 2011 was conducted across more than 10 provinces and autonomous cities, including Beijing, Chongqing, Guangxi, Guizhou, Heilongjiang, Henan, Hubei, Hunan, Jiangsu, Liaoning, Shaanxi, Shandong, Shanghai, Yunnan, and Zhejiang. Our sample from the CHNS 2011 includes 5884 households and 12,991 adults, which provides a large nationwide sample of the Chinese population in terms of major behavioral health and disease burden.

We restricted our analysis to those aged ≥18 years (*n* = 12,991) and focused on those with hypertension (*n* = 3645) in 2011. Excluding missing values, 3579 adults were included in the final dataset for analysis. Written informed consent was obtained from each participant before any data were collected. Note that more recent CHNS data have not yet been released for us.

### Measurement and definitions

We selected variables regarding knowledge about and medical history of hypertension, as well as demographics, socioeconomic status, and lifestyle behaviors from the adult household questionnaire in CHNS. We categorized geographic regions into three groups: Western China (Guangxi, Guizhou, and Chongqing), Eastern China (Shanghai, Beijing, Jiangsu, Liaoning, and Shandong) and Central China (Henan, Hubei, and Heilongjiang). Marital status was classified into currently married and single (i.e., never married, divorced, widowed, or separated). There were six a priori characteristics selected: residency (urban vs. rural), ethnicity (Han vs. minority), occupation (employed vs. unemployed), educational attainment **[**elementary school (≤6 years of education), middle school (6–9 years of education), high school or technical school (9–12 years of education), and college or above (> 12 years of education**].** Smoking status was defined as non-smoker (subjects who responded negatively to “have you ever smoked cigarettes?”), ex-smoker (subjects who responded positively to questions “have you ever smoked cigarettes?”, but negatively to “do you still smokes cigarettes?”;, and current smoker (as subjects who responded both positive answers to questions “have you ever smoked cigarettes?” and “do you still smokes cigarettes?”). Alcohol consumption status was defined as drinker (subjects replied “yes” to “do you regularly drink alcohol since last year?” and non-drinker. Medical insurance status was classified as insured vs. not insured. The physical examination, including height and weight, were measured by health care professionals. Weight was measured in light clothing to the nearest 0.1 kg on a calibrated beam balance, while height was measured to the nearest 0.1 cm using a portable stadiometer. Body mass index (BMI) was calculated as weight (kg) divided by the height squared (m^2^). Overweight and obesity were defined as a BMI of at least 24 kg/m^2^ and 28 kg/m^2^, respectively, based on the recommendations of the Working Group on Obesity in China [14].

### Assessment of hypertension and hypertension awareness, treatment, and control

The measurement and definition of hypertension was reported according to the 7th Chinese Joint National Commission guidelines [15]. A standard mercury sphygmomanometer was used by well-trained physicians to measure SBP and DBP on the right arm in triplicate after a 10-min seated rest. The mean of the three readings was calculated and used in all analysis. Hypertension was defined as having an average SBP ≥ 140 mmHg and/or an average DBP ≥ 90 mmHg, and/or a self-reported previous diagnosis of hypertension by a health care provider, and/or taking antihypertensive drugs currently. Awareness of hypertension status was defined as a self-report of any previous diagnosis of hypertension by a health care provider. Treatment of hypertension was defined as a self-reported use of antihypertensive medications at present. Control of hypertension was defined as having an average SBP < 140 mmHg and an average DBP < 90 mmHg while under pharmacological treatment for hypertension.

We compared our results on hypertension prevalence, awareness, treatment, and control rates with results from China Health and Nutrition Survey 2001 (CHNS 2001) and the International Collaborative Study of Cardiovascular Disease in ASIA (InterASIA 2000–2001). Detailed information on these two studies have been reported previously [16, 17]. In brief, CHNS 2001 study was conducted in 31 provinces, autonomous regions and municipalities throughout China. Stratified multistage cluster sampling was used to recruit participants and 141,892 participants ≥18 were analyzed as the final sample size. In the InterASIA study, stratified sampling method was used to select a nationally representative sample of population aged 35–74 years in China during 2000–2001, and 14,989 subjects were included in the analysis. These comparisons can provide evidence on the improvements in hypertension prevalence, awareness, treatment and control over the past 10 years and between different samples.

### Statistical analysis

Data were presented as mean ± SD for continuous and percentages for categorical variables according to gender, respectively. Differences between groups were tested using two-sample student t-tests for continuous variables and the Chi-square test for categorical variables. Multivariable logistic regression models were fit to explore the associations between relevant risk factors and hypertension awareness and treatment.

We also investigated the adjusted associations between independent variables and taking antihypertensive drugs among subjects who were aware of hypertension. Finally, characteristics and proportions of subjects by age groups were analyzed to identify the subpopulations that were more likely to take antihypertensive medications (“adherence”) in the subsample of participants who took treatments for hypertension. All the analysis was done using Stata 15.0 (StataCorp., 2017). *P* values were 2-tailed and *p* <  0.05 was considered to be statistically significant.

## Results

### General characteristics

The mean age of participants with hypertension was 60 years. About half of them were men (50.4%) and 58.5% were from rural areas. Table 1 presents their demographic characteristics, behavioral risk factors, and weight status. Rates of smoking and alcohol consumption were significantly higher in men than in women (*p* <  0.001). Women had a higher obesity (BMI ≥ 28 kg/m^2^) prevalence (22.6% vs. 18.4%) than men (*p* = 0.01).

**Table 1:** Demographic characteristics of Chinese adults with hypertension based on China Health and Nutrition Survey 2011

	All	Women	Men	*P*-value^**^
Sample size (N)	3579	1775	1804	
Age (year, mean ± SD)	60.0 ± 12.4	61.6 ± 11.7	58.5 ± 12.9	< 0.001
BMI (kg/m^2^)	25.3 ± 5.4	25.4 ± 5.2	25.2 ± 5.6	0.13
Residence (%)				
Urban	41.5	41.5	41.5	1.0
Rural	58.5	58.5	58.5	
Geographic region^b^ (%)				0.33
Western China	20.1	21.1	19.1	
Eastern China	49.4	48.6	50.3	
Central China	30.5	30.3	30.6	
Marital status^a^ (%)				< 0.001
Married	83.2	76.0	90.3	
Single	16.8	24.0	9.7	
Ethnicity				1.0
Han	92.0	92.0	90.0	
Minority	8.0	8.0	8.0	
Occupational status (%)				< 0.001
Employed	44.1	32.1	56.0	
Unemployed	55.9	67.9	44.0	
Health insurance (%)				
Insured	95.8	95.9	95.7	0.82
Not insured	4.2	4.1	4.3	
Education (%)				< 0.001
≤ Elementary school	48.2	60.8	35.7	
Middle school	27.4	22.4	32.4	
High school	17.1	13.0	21.1	
≥ College	7.3	3.8	10.8	
Smoking (%)				< 0.001
Non-smoker	65.9	94.3	38.0	
Ex-smoker	6.4	0.8	11.8	
Smoker	27.7	4.9	50.2	
Alcohol consumption (%)				< 0.001
Drinking	34.5	8.7	59.9	
Not drinking	65.5	91.3	40.1	
Weight status (%)				0.01
Normal weight (18.5 ≤ BMI < 24)	36.4	36.0	36.8	
Underweight (BMI < 18.5)	2.2	2.3	2.0	
Overweight (24 ≤ BMI < 28)	41.0	39.1	42.8	
Obese (BMI ≥ 28)	20.5	22.6	18.4	

Hypertension was defined as having an average systolic blood pressure BP ≥ 140 mmHg, diastolic blood pressure ≥ 90 mmHg, self-reported being previously diagnosed as hypertension by a physician or taking antihypertension drugs currently;

Non-smoker was defined as subjects who responded negatively to “have you ever smoked cigarettes?”; ex-smoker was defined as subjects who responded positively to questions “have you ever smoked cigarettes?” but negatively to “do you still smokes cigarettes?”; current smoker was defined as subjects who responded both positive answers to questions “have you ever smoked cigarettes?” and “do you still smokes cigarettes?”; drinking refers to subject who regularly drink alcohol since last year

^**^p-value was calculated from t-test for continuous variables and *chi-*square test for categorical variables

^a^Single includes never married, divorced, widowed and separated;

^b^Western China includes Guangxi, Guizhou and Chongqing; Eastern China includes Shanghai, Beijing, Jiangsu, Liaoning and Shandong; Central China includes Henan, Hubei, Henan and Heilongjiang;

### Awareness, treatment, and control of hypertension

**Fig. 1:**
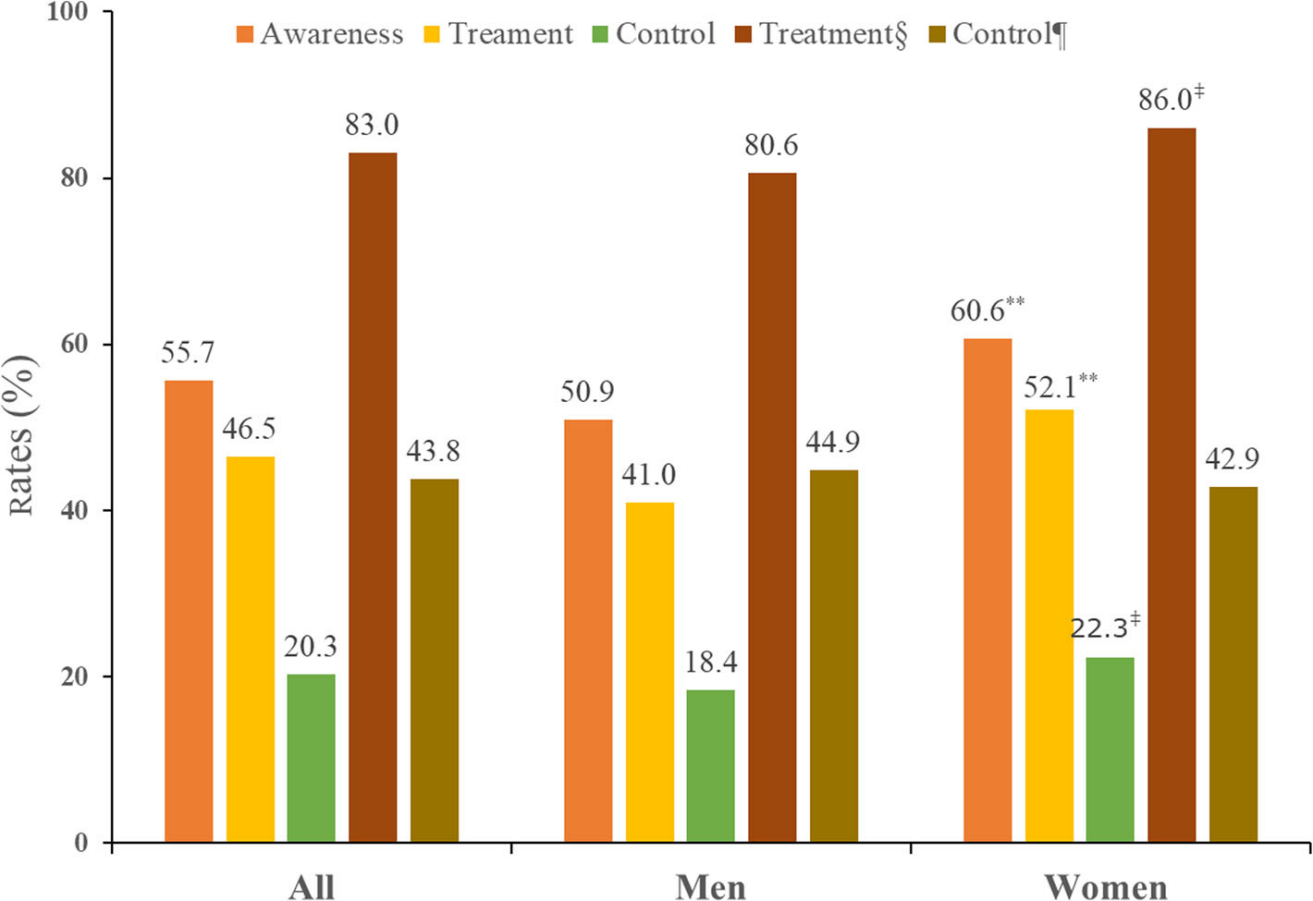
Rates of hypertension awarness, treatment, and control among Chinese adults with hypertension (*n* = 3579) by sex based on CHNS 2011

Overall, 27.6% of Chinese adults had hypertension in the sample, and the hypertension prevalence were 29.5 and 25.8% in men and women (*p* <  0.001), respectively. The estimated prevalence of awareness, treatment, and control were 55.7, 46.5, and 20.3% among Chinese adults with hypertension, respectively. The awareness, treatment and control rates among those with hypertension were higher in women compared with men (Fig. 1).

Of those who were aware of their hypertension status, 83.0% (*n* = 1664) received antihypertensive treatment. In addition, 43.8% (*n* = 728) of those who received treatment had their blood pressure under control (SBP/DBP < 140/90 mmHg).

Table 2 presents the proportions of hypertension outcomes in different subgroups. Older adults (≥ 65 yrs) had higher hypertension awareness and treatment rates compared to young adults (18–49 yrs). Hypertension awareness, treatment and control rates were higher in urban areas compared to rural areas and were higher in Eastern China compared to Central China. Participants who had a higher educational attainment (high school and above) were more likely to be aware of hypertension, and got treated and controlled. In contrast, participants who were ethnic minorities and obese were less likely to have controlled hypertension.

**Table 2:** Proportion^¶^ (%) of hypertension awareness, treatment, and control among Chinese adults with hypertension (HTN) based on CHNS 2011

	Awareness (1993)	Treatment (1664)		Control (728)	
		HTN	Awareness^a^	HTN	Treated^b^
Age group (years)					
18–49	36.2	25.9	71.7	12.7	49.0
50–64	58.1^**^	48.6^**^	83.6^**^	22.2^**^	45.7
65+	64.8^**^	56.8^**^	87.6^**^	22.8^**^	32.9
Residence					
Rural	48.0	38.4	80.0	13.5	35.2
Urban	66.6^**^	58.0^**^	87.0^**^	30.0^**^	51.7^**^
Geographic region^†^					
Central China	50.1	39.6	79.0	13.3	33.6
Western China	47.2	39.1	82.9	15.7	40.2
Eastern China	62.6^**^	53.8^**^	85.1^**^	26.6^**^	49.4^**^
Ethnicity					
Han	56.7	47.5	83.7	21.3	44.9
Minority	44.1^**^	35.0^**^	79.4	9.1^**^	26.0^‡^
Marital status^*^					
Unmarried	58.9	49.8	84.5	19.6	39.5
Single	55.0	45.8	83.3	20.5	44.7
Occupational status					
Unemployed	64.5	56.4	87.4	24.4	43.3
Employed	44.6^**^	34.0^**^	76.3^**^	15.2^**^	44.7
Health insurance					
Not insured	51.3	42.0	81.8	19.3	46.0
Insured	55.9	46.7	83.6	20.4	43.7
Education					
≤ Elementary school	54.2	45.0	82.9	16.0	35.6
Middle school	52.9	43.8	82.9	21.2^‡^	48.4^**^
High school	60.0^§^	51.6^‡^	86.1	27.1^**^	52.5^**^
≥ Collage	65.9^**^	54.8^‡^	83.1	29.9^**^	54.5^**^
Smoking					
Non-smoker	58.4	50.1	85.7	21.7	43.3
Ex-smoker	68.4^‡^	55.3	80.8	28.5^§^	51.6
Smoker	46.3^**^	36.0^**^	77.8^**^	15.3^**^	42.6
Alcohol consumption					
Not drinking	58.7	50.9	86.8	21.5	42.1
Drinking	50.0^**^	38.1^**^	76.0^**^	18.2^§^	47.9
Weight status					
Normal weight (BMI < 24)	50.7	40.5	79.8	20.2	50.0
Overweight (24 ≤ BMI < 28)	58.6^**^	49.6^**^	84.8^§^	21.8	43.8
Obese (BMI ≥ 28)	59.4^**^	51.6^**^	83.5^‡^	17.7	34.4^**^

^¶^3579 adults with hypertension;

Hypertension was defined as having an average systolic blood pressure ≥ 140 mmHg, diastolic blood pressure ≥ 90 mmHg, self-reported being previously diagnosed as hypertension by a physician or taking antihypertension drugs currently;

Non-smoker was defined as subjects who responded negatively to “have you ever smoked cigarettes?”; ex-smoker was defined as subjects who responded positively to questions “have you ever smoked cigarettes?” but negatively to “do you still smokes cigarettes?”; current smoker was defined as subjects who responded both positive answers to questions “have you ever smoked cigarettes?” and “do you still smokes cigarettes?”; drinking refers to subject who regularly drink alcohol since last year

Awareness of hypertension status was defined as a self-report of hypertension diagnosed by a doctor before, or taking any antihypertensive drugs;

Control of hypertension was defined as blood pressure (SBP/DBP) < 140/90 mmHg;

^*^Single includes never married, divorced, widowed and separated;

^†^Western China includes Guangxi, Guizhou and Chongqing; Eastern China includes Shanghai, Beijing, Jiangsu, Liaoning and Shandong; Central China includes Henan, Hubei, Henan and Heilongjiang;

*Chi-square* test were performed ^§^*p* < 0.05, ^‡^*p* < 0.01, ^**^p < 0.001

^a^ Treatment among subjects who were aware of their hypertension;

^b^ Control among participant who took antihypertension medications;

### Hypertension prevalence, awareness, treatment in CHNS 2001 and InterASIA 2000–2001

Overall, hypertension prevalence in CHNS 2001 was 18.0%. Hypertension awareness, treatment, and control rates were 24.0, 20.0 and 4.5% among subjects with hypertension in China, respectively. Of these 24.0% individuals who were aware of their condition, 78% were receiving prescribed antihypertensive medications, and 24% of those receiving treatment were adequately controlled. The overall hypertension prevalence was 26.5%, and hypertension awareness, treatment, and control rates were 43.0, 29.9, and 7.7%, respectively in InterASIA study. Of those who were aware of their hypertension, 61.5% received treatment, and 29.1% of those who received treatment had their blood pressure controlled (SBP/DBP < 140/90 mmHg). **(**Table 3**)** These hypertension rates were lower than those in our study.

**Table 3:** Comparison of average blood pressure, hypertension prevalence, awareness, treatment and control across China Health and Nutrition Survey (CHNS) 2011, CHNS 2001 and the International Collaborative Study of Cardiovascular Disease in ASIA (InterASIA 2000–2001) among Chinese adults

	CHNS 2011	CHNS 2001 [16]	InterASiA (2000–2001) [17]
Blood pressure^a^	SBP/DBP	SBP/DBP	SBP/DBP
All subjects	143/89	N/A	149/90
Those took treatment	143/87	N/A	148/89
Those did not take treatment	143/90	N/A	149/91
Hypertension profile (%)			
Prevalence	27.6	18.0	26.5
Awareness	55.7	24.0	43.0
Treatment^a^	46.5	20.0	29.9
Treatment^b^	83.0	78	61.5
Control^a^	20.3	4.5	7.7
Control^c^	43.8	24	29.1

Control of hypertension was defined as SBP/DBP < 140/90 mmHg among subjects receiving pharmacological treatment;

N/A indicated data not specified

*SBP* Systolic blood pressure, *DPB* Diastolic blood pressure

^a^among subjects with hypertension;

^b^among subjects who were aware of their hypertension;

^c^among subjects who took antihypertensive medications;

### Factors associated with hypertension awareness and treatment

Table 4 presents findings from multivariate logistic regression that identified factors associated with hypertension awareness, treatment, and control. Women were more likely to be aware of their hypertension than men, with an OR of 1.37 (95% CI, 1.12–1.66). Rural residency, minority ethnicity, and unemployment were negatively associated with hypertension awareness. Adults who were overweight and obese were more likely to be aware of their hypertension compared to those with normal weights. Factors associated with treatment of hypertension were similar to those related to hypertension awareness except for smoking status and drinking. Smoking status was not associated with treatment of hypertension. Current drinkers were less likely to be taking antihypertensive medication (OR, 0.53; 95% CI, 0.39–0.72).

**Table 4:** Multivariable logistic regression analysis of factors associated with hypertension awareness, treatment and control among Chinese adults with hypertension (*n* = 3579) in CHNS 2011

	Awareness	Treatment^a^	Control	Control^b^
Women (vs. men)	1.37 (1.12–1.66)	0.94 (0.65–1.34)	1.32 (1.04–1.68)	1.17 (0.88–1.57)
Age group (years)				
18–49 (ref)				
50–64	2.34 (1.92–2.85)	1.87 (1.32–2.65)	1.87 (1.43–2.44)	1.33 (0.92–1.92)
65+	3.13 (2.45–3.99)	2.57 (1.65–4.02)	1.84 (1.34–2.52)	1.06 (0.70–1.62)
Residence				
Urban (vs. rural)	1.75 (1.50–2.04)	1.50 (1.14–1.97)	2.16 (1.79–2.60)	1.79 (1.43–2.24)
Geographic region^c^				
Central (ref)				
Western	0.92 (0.75–1.12)	1.35 (0.93–1.96)	1.33 (1.01–1.75)	1.33 (0.95–1.88)
Eastern	1.50 (1.27–1.77)	1.52 (1.14–2.01)	2.12 (1.72–2.63)	1.78 (1.37–2.31)
Ethnicity (vs. Han)				
Minority	0.74 (0.57–0.96)	0.78 (0.49–1.26)	0.47 (0.31–0.73)	0.52 (0.31–0.86)
Occupational status (vs. unemployed)				
Employed	0.78 (0.66–0.93)	0.74 (0.54–1.00)	0.92 (0.74–1.15)	0.96 (0.72–1.26)
Marital status^d^ (vs. single)				
Married	1.09 (0.89–1.34)	1.27 (0.89–1.83)	1.11 (0.87–1.43)	1.16 (0.86–1.57)
Health insurance (vs. not insured)				
Insured	1.32 (0.93–1.87)	1.44 (0.77–2.68)	1.08 (0.70–1.66)	0.90 (0.52–1.55)
Weight status				
Normal weight ((BMI < 24) (ref)				
Overweight (24 ≤ BMI < 28)	1.41 (1.20–1.66)	1.52 (1.15–2.01)	0.96 (0.79–1.16)	0.83 (0.65–1.05)
Obese (BMI ≥ 28)	1.57 (1.29–1.92)	1.99 (1.39–2.84)	0.77 (0.60–0.98)	0.56 (0.42–0.76)
Education				
≤ Elementary school (ref)				
Middle school	1.11 (0.92–1.33)	1.08 (0.78–1.50)	1.39 (1.11–1.74)	1.57 (1.20–2.06)
High school	1.34 (1.07–1.67)	1.42 (0.97–2.08)	1.65 (1.28–2.12)	1.53 (1.13–2.07)
≥ College	1.69 (1.23–2.32)	1.04 (0.64–1.71)	1.77 (1.26–2.48)	1.80 (1.20–2.70)
Smoking status				
Non-smoker (ref)				
Ex-smoker	1.62 (1.16–2.24)	0.75 (0.46–1.23)	1.47 (1.04–2.09)	1.23 (0.80–1.88)
Smoker	0.86 (0.70–1.04)	0.83 (0.59–1.18)	0.81 (0.63–1.05)	0.83 (0.61–1.14)
Alcohol consumption				
Drinking (vs. not drinking)	0.95 (0.79–1.13)	0.53 (0.39–0.72)	0.96 (0.77–1.19)	1.03 (0.78–1.35)

Hypertension was defined as having an average systolic blood pressure ≥ 140 mmHg, diastolic blood pressure ≥ 90 mmHg, self-reported being previously diagnosed as hypertension by a physician or taking antihypertension drugs currently;

Non-smoker was defined as subjects who responded negatively to “have you ever smoked cigarettes?”; ex-smoker was defined as subjects who responded positively to questions “have you ever smoked cigarettes?” but negatively to “do you still smokes cigarettes?”; current smoker was defined as subjects who responded both positive answers to questions “have you ever smoked cigarettes?” and “do you still smokes cigarettes?”; drinking refers to subject who regularly drink alcohol since last year

Awareness of hypertension status was defined as a self-report of hypertension diagnosed by a Physician before, or taking any antihypertensive drugs;

Control of hypertension was defined as blood pressure (SBP/DBP) < 140/90 mmHg;

^a^Treatment among subjects who were aware of their hypertension;

^b^control among participant who took antihypertension medications;

^c^Western China includes Guangxi, Guizhou and Chongqing; Eastern China includes Shanghai, Beijing, Jiangsu, Liaoning and Shandong; Central China includes Henan, Hubei, Henan and Heilongjiang

^d^Single includes never married, divorced, widowed and separated;

### Factors associated with hypertension control

Hypertension was better controlled in women (1.32, 1.04–1.68) compared with men, and in older (≥ 65 years old) (1.84, 1.34–2.52) compared to young or mid-aged adults People in urban areas (2.16; 1.79–2.60) and those from Eastern China (2.12; 1.72–2.63) were more likely to have controlled hypertension. In contrast, minority ethnicity (0.47; 0.31–0.73) and obese (0.77; 0.60–0.98) participants were less likely to have their hypertension controlled. Among participants taking medication for hypertension, those in an urban area, located in Eastern China, and with higher educational attainment had higher odds of hypertension control, whereas minorities (0.52; 0.31–0.86) and people with obesity (0.56; 0.42–0.76) were less likely to have their blood pressure controlled.

## Discussion

This study provides important evidence on the burden of hypertension in China. Over a quarter (27.6%) of Chinese adults had hypertension. Among those with hypertension, only 55.7% were aware of their condition, 46.5% were taking antihypertensive medication to lower their blood pressure, and only 20.3% achieved blood pressure control. Being female, having an older age, living in urban areas or the Eastern region, having a high educational attainment, not drinking alcohol, and not currently smoking were associated with higher rates of hypertension awareness, treatment, and control.

Our findings on hypertension awareness, treatment, and control are consistent with a similar previous study [18]. The hypertension awareness and treatment rates in the present study were more than two times greater than those seen a decade ago [16] and the control rate was substantially higher than that reported in the InterASIA study [17]. This is likely attributable to joint efforts paid by public health practitioners and health professionals in this time period.

In a systematic review based on data from 35 countries, hypertension treatment (22%) and control rates (5.3%) in China in 2009–2010 were lower than the average level of many developing countries, such as Indonesia (25% of treatment rate) and India (10.1% of control rate), respectively [19]. Our study showed that hypertension treatment among those who aware of hypertension (83.0%) in 2011 was slight lower than the global average level of 87.5% in 2009, but the control rate among those were treated (43.8%) were higher than global average (32.5%) [20, 21]. The overall hypertension awareness (55.7%), treatment (46.5%) and control (20.3%) rates among subjects with hypertension in our study were lower than those in developed countries, such as in the US (80.7, 72.5, and 50.1%, respectively), and in Germany (82.3, 71.8 and 51.2%, respectively) around 2010 [22, 23]. The abovementioned discrepancies of hypertension rates between China and developed countries may due to the limited access to primary health care facilitates, and lack of professional health personnel and essential antihypertensive medications in China.

Our multivariate logistic regression analysis identified several factors being associated with awareness, treatment, and control among all participants with hypertension. Consistent with several previous studies [24–26], we found that women were more likely to be aware of their hypertension and achieve hypertension control than men. Older individuals (≥ 65 years) and urban residents had higher hypertension awareness and control rates. A possible reason is that urban residents and the elderly may be more careful about their health [9, 27, 28]. Participants in Eastern China had higher rates of hypertension awareness, treatment and control rates compared to those in Central China, This could be explained by the facts that Eastern China residents have high socioeconomic status, and higher socioeconomic status associates with easier access to basic healthcare facilities and primary health care practitioners [29, 30].

Being ethnic minority was associated with lower rates of hypertension awareness, and control. This difference may be attributed to variations in socioeconomic status, culture, lifestyles between ethnic minorities and the majority Han ethnicity [31]. China has a diversity of ethnic groups and cultures as well as heterogeneity of socioeconomic levels. Compared with Han, ethnic minority was associated with lower economic status and health literacy, and has a number of barriers, such as limited health care access and utilization, distrusting in clinical community, poor adherence to medications, and language/cultural barriers. Employed participants were less aware of their hypertension status than unemployed subjects. This may due to that employed subjects usually were overall healthy thus had less frequency of body check-ups.

An education level of high school and above was found to be associated with higher hypertension awareness, treatment and control rates. This mainly attribute to that subjects with higher educational level were more likely to seek medical care, and more likely to adherent to instructions/medications than other [32, 33]. However, our result was consistent with a study based on data collected from adults in Southern China [34] but was different from what was reported by the CHPSNE study [35]. The disparity may reflect differences in the study sample and treatment of hypertension in these studies.

Being overweight and obesity were positively associated with higher rates of hypertension awareness and treatment, which was consistent with several other studies [36, 37]. However, only obesity was associated with a lower control rate than subjects with normal weight. This could be explained by poor adherence to medical treatment among subjects with a suboptimal body weight [38, 39]. We assumed that subjects with a suboptimal body weight status (overweight/obesity) would be less likely to pay attention to their health conditions than those with a normal weight status, so they might had poor adherence to medical therapies prescribed by physicians.

In addition, our study found that the awareness and control rates were greater among former smokers and lower among current smokers when compared with non-smokers. This could be explained that those who used to smoke were more health conscious and, thus, be more adherent to their medication than non-smokers [40]. The exact reasons behind this association require further investigations which beyond the scope of the current study.

Increased proportion of hypertension awareness, treatment, and control has been made during the past decades, but the control of hypertension remains at unacceptably low level. Our findings indicates that only one fifth (20.3%) of participants with hypertension had their blood pressure controlled. Mainly, three aspects of factors contributed to the poor control rate. First, the screening and payments within the health system. In much of China, screening is simply a process and does not lead to follow-up. Besides, a zero-profit drugs policy introduced by health reforms incentives doctors to prescribe more expensive drugs rather than the essential drugs [10]. Second, lack of qualified primary-care physicians who could proscribe appropriate antihypertensive drugs. Third, unhealthy lifestyle factors, such as obesity, heavy drinking and smoking, could result in incompliance to prescribed medications. Several effective and feasible strategies could be carried out to improve hypertension control in China. First, strengthening health systems by integrating hypertension screening and follow-ups into routine primary care practice, removing financial barriers to health care, and improving access to essential and affordable medications. Second, programs of professional education should be implemented to improve primary care physician’s prescription habits, physician’s knowledge of and willingness of adhere to new hypertension treatment guidelines, such as the use of appropriate combined therapy from evidence-based medicine. Finally, comprehensive health education about dangers of hypertension and strategies to modify the undesirable lifestyles/habits must be given to patients to promote the importance of management and monitoring of blood pressure, in order to obtain the optimal control of hypertension.

Our study has several limitations. First, cross-sectional data analysis does not support causal inferences between risk factors and hypertension outcomes. Second, blood pressure was not measured on separate occasions. Since the definition of hypertension was partly based on the blood pressure measurement, the absolute hypertension burden might be overestimated. Third, the CHNS did not capture non-pharmacological treatment strategies such as healthy diet, adequate exercise, stress reduction, and sufficient amounts of potassium and magnesium. Fourth, the age range in CHNS (≥18 years old) and the InterASIA (35–74 years old) was different, so there is potential bias due to this age difference when doing comparison and interpretation. However, the large study sample size and various risk factors that examined in the analysis models stands the strengths of this study.

## Conclusion

In conclusion, hypertension awareness, treatment, and control rates have increase significantly since 2001 in China, but the treatment and control rates remain relatively low especially among certain subgroups, such as in younger adults, those live in rural areas and underdeveloped regions, and those with unhealthy lifestyles. Substantial efforts—blood pressure screening in clinical practice, standardized educational programs for healthcare providers, and modification of unhealthy lifestyle factors in patients—are needed to improve hypertension treatment and control in China.

## Data Availability

The datasets used and/or analyzed during the current study are not publically available. Permissions could be obtained/required from the corresponding author on a reasonable request.

## Abbreviations

OR: odds ratio
95% CI: confidence interval
SD: Standard deviation
BMI: Body mass index
SBP: Systolic blood pressure
DBP: Diastolic blood pressure
HTN: Hypertension
CHNS: China Health and Nutrition Survey
